# Serious engagement in sport and health benefits among Korean immigrants in the USA

**DOI:** 10.3402/qhw.v11.31340

**Published:** 2016-08-03

**Authors:** Junhyoung Kim, May Kim, Karla A. Henderson, Areum Han, Se-Hyuk Park

**Affiliations:** 1 Recreation, Parks, and Leisure Services Administration, Central Michigan University, Mount Pleasant, MI, USA; 2 Department of Physical Education, Anam-dong, Seongbuk-gu, Seoul, Korea University, Seoul, South Korea; 3 Parks, Recreation, and Tourism Management, North Carolina State University, Raleigh, NC, USA; 4 Korea University Center for Curriculum and Institute of Studies, Korea University, Seoul, South Korea; 5 Department of Sports Sciences, Seoul National University of Science and Technology, Seoul, South Korea

**Keywords:** Acculturation, benefits, Korean immigrants, serious leisure, sports clubs

## Abstract

There is a dearth of information pertaining to ethnicity and serious leisure among immigrants. The purpose of our study was to explore the health benefits of serious engagement in sports among Korean immigrants who are part of club activities. Using semi-structured in-depth interviews, we identified three themes associated with the benefits of serious leisure: (a) coping with acculturative stress, (b) creating ethnic strength, and (c) personal benefits. Participants gain personal and social benefits by pursuing leisure activities in a serious manner within their ethnic in-group.

According to social identity theory, when individuals interact with others who have different cultural backgrounds, they easily acknowledge inherent cultural and ethnic differences, and they tend to express both in- and out-group bias (Tajifel & Turner, 1986). Thus, individuals have a tendency to form and develop a sense of friendship with other in-group members, who maintain similar cultural understandings (Shinnar, 2008). Several researchers have found that engaging with in-group members enables individuals to establish a positive social identity and self-concept (e.g., Brewer, 2007; Worchel, 2005). Individuals who maintain a strong sense of in-group favoritism tend to express negative attitudes and perceptions towards out-group members (Brewer, 2007; Rustemli, Mertan, & Ciftci, 2000).

In the context of sports and recreational activities, individuals may preserve their in-group bias and favoritism, and they tend to engage in activities with in-group members. Studies have provided evidence that in-group members of a sports club from the same ethnic minority exhibited greater feelings of belonging and in-group bonding than members of a sports club who were ethnically distinct (e.g., Elling, Knoppers, & De Knop, 2001; Kim & Kim, 2013). By being involved in a sports club with in-group members of the same ethnic minority, they gained feelings of connectedness and created a social support system for each other—both inside and outside of the context of sports (Walseth, 2006).

Immigrants can experience dynamics of both intra- and intergroup relations through participation in leisure activities. Kim (2012) found that female Korean immigrants living in the United States enhanced their sense of cultural and ethnic understandings and developed friendships with out-group members through leisure activities. They also tended to engage in recreational activities with in-group members because of cultural and ethnic similarities (Kim, 2012).

Kim, Heo, and Kim (2014) investigated a group of Korean immigrants who organized their own sports club with in-group members and maintained cultural identification and membership. They suggested that immigrants possessed in-group membership—and that as part of sports clubs, they may express serious involvement in certain recreational activities. Stebbins (1982, 1992) used the term “serious leisure” to explain those who are committed to an activity and who center their lives on the special skills, knowledge, and experience that their particular activity demands. Heo and Lee (2007) investigated serious-leisure experiences among Korean international students who were part of a basketball club and found that participants formed intimate in-group relationships through sports.

Although a few studies have explored the relationship between serious involvement in an activity and ethnicity (Heo & Lee, 2007; Lee, Dunlap, & Scott, 2011), a dearth of information exists about ethnicity and serious leisure among immigrants who are members of ethnic sports-club activities. The purpose of our study was to explore experiences from the Perspectives of serious leisure and capture the health benefits of participation in recreational activities—among Korean immigrants who are part of club activities. To respond to this purpose, literature was examined pertaining to the immigrant experience, in-group activity, serious leisure, and sports.

## In-group activities and immigrants/members of ethnic minorities

There are mixed findings about the positive and negative effects of immigrants participating in sports. Walseth and Fasting (2004) emphasized the value of sports participation for social and cultural integration—among immigrants and members of ethnic minorities. For example, female Korean immigrants who participated in various recreational activities enhanced their cultural, ethnic understanding and developed out-group friendships (Kim, 2012). Despite the value of participating in sports, immigrants and members of ethnic minorities experienced social exclusion, racial differences, and cultural resistance from host individuals in the sports context, which resulted in a lack of engagement in sports (Bradbury, 2011; Spaaij, Magee, & Jeanes, 2014).

Some immigrants have organized their own sports teams or clubs (i.e., in-group sports) because of in-group members’ more positive views and attitudes, compared to out-group members (Sidanius, Pratto, & Mitchell, 1994; Toosi, Babbitt, Ambady, & Sommers, 2012). Elling et al. (2001) suggested that in-group members tend to demonstrate their performances in sports and maintain cultural identities and membership through in-group sport contact. For example, members of ethnic minorities in Norway established their own cultural organizations to reinforce and maintain their sense of ethnic and religious belonging by participating in sports (Elling & Claringbould, 2005; Walseth, 2006). By interacting with in-group members of their own sports club, they created a support system and engaged in in-group bonding, resulting in feelings of belonging and in-group cohesion (Walseth, 2006).

Immigrants usually consider themselves representatives of their ethnic group and tend to preserve their cultural perspective by interacting with in-group members (Hopkins, Greenwood, & Birchall, 2007; Safdar, Lay, & Struthers, 2003). For example, Iwasaki and Barlett (2004)) discovered that Aboriginal individuals with diabetes who participated in activities related to their culture found social, psychological, and cultural empowerment as a result. Afghan immigrants created and participated in activities related to their culture, which fostered social bonding (Stack & Iwasaki, 2009). Research also indicates that immigrants desire to maintain their ethnic identity and cultural heritage by participating in activities connected with their culture (Kim, Kleiber, & Kropf, 2001). However, there have been few studies about ethnicity, sports, and serious leisure.

## Serious involvement in sports

Stebbins (1992) proposed six distinct qualities of serious leisure that distinguish it from casual leisure: (a) perseverance, (b) significant effort, (c) career development, (d) durable benefits, (e) enablement of the expression of self and identity, and (f) a unique ethos. Stebbins (1996) identified various benefits of serious leisure, including personal enrichment, self-actualization, self-expression, a positive self-image, self-gratification, recreation, and financial returns. In addition, he identified social rewards, such as social attraction, group accomplishment, and contribution toward the development of the group.

A growing body of literature provides evidence that serious-leisure participation can lead to psychological and physical benefits throughout an individual's lifespan (Gould, Moore, Karlin, Gaede, Walker, & Dotterweich, 2011; Heo, Lee, McCormick, & Pederson, 2010; Kim, Yamada, Heo, & Han, 2014). For example, in their study on Senior Games participants, Heo, Stebbins, Kim, and Lee (2013) found that the level of involvement with serious leisure was positively related to life satisfaction and health. In addition, Gallant, Aria, and Smale (2013) made the following proposal: Through participation in serious leisure, an individual can discover a lifestyle that contributes to his or her community, human fulfillment, and the maximization of human potential.

Through participation in sports activities, serious leisure has been mainly studied to understand the behaviors of sports participants and to identify the potential benefits of participation. The concept of *serious leisure* has been examined in a variety of sports-related studies, involving white-water kayakers (Kane & Zink, 2004), runners (Shipway & Jones, 2008), older golfers (Siegenthaler & O'Dell, 2003), youths (Siegenthaler & Gonzalez, 1997), cyclists (Herman, 2015), and mountain climbers (Dilley & Scraton, 2010). For example, Tae kwon do participants in the serious-leisure cluster showed higher levels of life satisfaction and subjective well-being than those who engaged in this activity less seriously (Kim, Dattilo, & Heo, 2011).

Although an increasing number of studies in sports and serious leisure have been conducted, few studies have investigated involvement in sports that are related to serious leisure—from immigrant perspectives. However, several studies have revealed that acculturation is positively associated with involvement in sports and physical activities among Asian immigrants (Kim et al., 2014; Lee, Sobal, & Frongillo, 2000). Furthermore, Lee et al. (2011) provided evidence that Korean immigrants who engaged in serious leisure through basketball and soccer games with members of different racial groups came to embrace different cultural values and exhibited enhanced self-identification. Our study was designed to add to the literature about the benefits that one can receive by participating in sports-club activities—and the influence of serious leisure that is related to in-group sports-club participation.

## Method

This study used semi-structured in-depth interviews, as this method is most appropriate for exploring the benefits of in-group sports participation among immigrants (Kim et al., 2014; Kim, Kim, Han, & Chin, 2015). This method enabled us to explore the serious-leisure experience in the context of in-group sports clubs.

### Participants

To identify in-group participants, this study used criterion-based sampling. The criteria for identifying and selecting participants were as follows: (a) legal immigrant status, (b) current membership in a Korean sports club, (c) self-admittance as a serious-leisure participant, (d) no participation in out-group sports as serious leisure, and (e) being 18 years of age or older.

For this study, we sampled members of sports clubs composed of Korean immigrants. An institutional review board of universities approved this study. Participants were informed that the study was confidential and that they could withdraw at any point. The study participants voluntarily enlisted, and only their last names were used for identification.

There were 16 total participants: 10 males and 6 females. They ranged in age from 34 to 64 years. The average length of time since they had immigrated was 21 years. Fourteen participants were married, and only one participant had a grandchild. All of the participants had college degrees or higher. The average length of time they had engaged in their sports club was 12 years. Out of the 16 participants, 10 were members of a badminton club, 3 belonged to a tennis club, and the others were members of a table tennis team. All of them had some sort of previous experience playing these sports before immigrating; in particular, the male participants had been avid sports participants in Korea. After immigrating, female participants had fewer opportunities to participate in sports because of their challenges with adapting after immigration. The number of interviews was based on the principle of data saturation (Guest, Bunce, & Johnson, 2006). Interviews were conducted and coded until no new themes or categories emerged.

### Data collection procedure

The research team gathered information about practice schedules and locations for club activities in the northwest USA. We used three sports clubs and acquired permission from the president of each club. The clubs provided tennis, table tennis, and badminton activities. Each club had about 25–50 members. We briefly introduced the study's purpose before the practice session commenced. Additionally, club presidents passed our contact information on to those who intended to participate. In turn, the participants contacted us directly to confirm their interest in the study.

Interviews were conducted at locations that were most convenient for the participants. Each interviewee was asked about his or her language preference; all preferred to speak Korean. The interviews lasted between 50 and 120 min, and they were recorded and transcribed with the participants’ permission.

As suggested by Legard, Keegan, and Ward (2003), we used content-mapping and content-mining questions. Content-mapping questions were used to widen the participants’ perspectives, widen thoughts and experiences, and share comprehensive coverage. Among the content-mining questions, we amplified probes and clarification, which allowed us to gain clarity and obtain detailed descriptions of experiences (Legard et al., 2003). Examples of questions were as follows:

- “Why did you participate in this activity?”
- “What benefits did you experience when you participated in this activity?”
- “Did you face any challenges in participating in this activity because you are Korean?”

### Data analysis

We incorporated McCracken's data analysis (1988), which is composed of five steps. To capture detailed, emerging themes and subthemes, we used the constant-comparison method, which allowed us to simultaneously code and analyze data—and to create domains of concepts.

In the first step, we procured raw data and read transcripts, making notations in the margins. The main task was to extract participants’ important experiences related to serious-leisure benefits from the transcripts. In the second step, observations from the first step were developed into Preliminary and descriptive categories. To identify whether preliminary categories were consistent among the interviews, we compared transcripts. In the third step, the preliminary codes presented by each researcher were scrutinized to identify connections—as well as similar and/or different patterns. In the fourth step, we reflected on clusters of ideas related to the preliminary codes and determined basic themes. Finally, in the fifth step, we examined and delineated the core themes and subthemes and selected data examples to support those themes.

This study used three strategies to enhance the trustworthiness of the findings. First, each researcher contributed expertise and experience in qualitative work, as emphasized by Elliott and Timulak (2005). The research team captured the findings with quotes and illustrative examples, based on thoughtful consideration and consensuses. Second, to increase the accuracy of the transcriptions, we applied the back-translation process. According to guidelines for back-translation developed by Suh, Kagan, and Strumpf (2009), the research team invited two bilingual graduate students to validate the accuracy of the translation of each transcription. Third, the researchers initiated member checking with a summary of identified themes and interpretations. Of the 16 participants, 11 participants voluntarily participated in the member-checking process and indicated that they were satisfied with the interpretations.

## Findings

Participants shared their experiences related to their participation in sports with in-group members. Based on their statements and experiences, the analyses showed evidence that participants manifested many of the serious-leisure qualities proposed by Stebbins (1982, 1992). The data indicated that the participants exhibited the qualities of serious leisure, such as perseverance, significant effort, the creation of unique cultural worlds, cultural membership and identity, and positive benefits.

For example, participants experienced challenges such as physical fatigue, injury, and pressure associated with their participation in sports-club activities. To overcome these challenges, participants persevered and achieved personal goals through sports-club activities. Participants exerted significant efforts to master their skills and techniques and continually pursued personal goals related to sports-club activities. In the context of in-group sports-club activities, participants shared their positive experiences with other Korean immigrants and created unique, cultural worlds to generate emotional and social support for one another. As team members, participants enhanced their cultural membership and identity.

Based on the identified qualities of serious leisure, we identified three salient themes associated with serious-leisure benefits: (a) coping with acculturative stress, (b) creating ethnic strength, and (c) personal benefits. These identified themes indicated that serious engagement in sports can contribute to social and psychological benefits.

### Coping with acculturative stress

All participants experienced a variety of adaptation challenges that entailed acculturative stress, such as language barriers, a lack of cultural understanding, racial discrimination, and limited social networks. They indicated that playing sports with other Koreans helped them cope with acculturative stress because they shared similar cultural values and beliefs. When they played sports with other ethnic groups, they had some difficulty developing strategies and communicating because of a lack of English skills. As Ha (a 34-year-old male participant) said:

I could not share my strategic plans with Americans because of my English skills, and it was so stressful to play badminton with them because of the language barrier. After I created a Korean badminton club, it was so much fun because I did not have any communication problems with others.

Almost all of the participants mentioned that they reduced their level of acculturative stress by obtaining social and emotional support through sports clubs. They believed that sports provided a positive atmosphere where they could preserve their cultural identity and create in-group bonding. For example, Yo (a 34-year-old female participant) said that the emotional and social support from her table tennis team helped relieve the stress generated from adapting to a new society.

In addition, some of them felt that their connections and sense of belonging with in-groups served as a buffer against acculturative stress. For example, Lee-J (a 35-year-old male participant) said that he used club activities as a mental escape from work—and as a means to escape from negative experiences he associated with trying to fit in. Hye (a 36-year-old female participant) experienced a sense of loneliness and organized a table tennis club because she realized that many Korean females similarly felt a lack of social support and consequently felt depressed. In a similar vein, Yang (a 44-year-old male participant) stated:

I got along with members of other ethnic groups when I played badminton. However, I felt a strong sense of belonging and friendship with other Korean players because we had a strong connection and possessed similar cultural values and beliefs … This club helped me a lot in coping with adaptation challenges.

Most female participants reported that they had been given few opportunities to play sports and had experienced social exclusion from members of other ethnic groups, which resulted in acculturative stress. After becoming members of sports clubs with other Korean members, these women believed that they utilized leisure resources well and participated in various intramural sporting events to compete with others. Such participation helped them become physically active and energetic. Yo (a 34-year-old female participant) stated:

Even though I saw some Americans play [table tennis], I could not join the game because I just felt that I was different from them … After joining the club, my [Korean] friends and I enjoyed competing with other teams and being more physically active.

She also mentioned that practicing sports helped her reduce acculturative stress.

The examples above showed that serious-leisure experience helped participants deal with acculturative stress and generate social and emotional support. In particular, through playing sports, participants built a sense of belonging and ethnic bonding that served as a buffer against acculturative stress.

### Creating ethnic strength

Creating ethnic strength was an additional theme that participants gained through in-group sports-club participation. By participating in sporting events hosted by the Korean community, most participants believed that they expanded they strengthened their ethnic identity because it was a unique opportunity for them to share cultural aspects of their origin country, such as language, food, and cultural values. They participated in Korean-American sports festivals every year at the state and national levels, and they engaged in ethnic-group bonding and culturally connected with members of other Korean teams. For example, as Kim-J (a 34-year-old male participant) said:

I participated in biannual Korean-American national and state sports festivals and competed with other Korean immigrants … I made friends with those who came from other states. We expect to participate in this sporting event for Korean immigrants every year. It is fascinating!

Participants appeared to experience a sense of ethnic empowerment and cultural strength. The sports festivals also offered a variety of traditional Korean games, food, entertainment, and plays, which might help create special bonds among Korean immigrants.

Similarly, Yang (a 44-year-old male participant) described how these sports festivals expanded his social network and interpersonal connections:

This is not just a sports competition. It is an ethnic union and a cultural event. I play with other Korean teams, and regardless of the results, we became good friends and expect to play again at the next event.

Some participants believed that they created a strengthened community by competing with sports teams from other ethnic groups. This competition helped them strengthen their ethnic identity and membership. When they played against teams with members from other ethnic groups, they said that they became much more competitive and focused on winning the game. This competitive orientation likely came from the belief that their team was representative of their own ethnicity. By contrast, participants sought to focus on enjoying the game when they played with in-group teams from their own ethnicity. For example, Lee (a 48-year-old male participant) stated:

I took the tennis match very seriously when I competed with others [from other ethnic groups]. The reason was because my team wanted to demonstrate the power of Koreans; in particular, when I played with Japanese-American teams, it was so brutal because of the history of the two countries.

He also indicated that historical facts influenced their attitudes toward (and preparation for) games. When they won, his team members were proud of their nation of origin.

According to the participants’ statements and experiences, serious involvement in sports provided opportunities for them to build and expand ethnic cohesion and strength. In particular, through competitive sports with other ethnic groups, participants focused more on competition, rather than enjoyment, and strengthened their cultural identity and membership. They also described personal benefits from this serious participation.

### Gaining personal benefits

All of the participants obtained personal benefits through serious participation in sports-club activities. These benefits included enhanced self-esteem and confidence, a sense of self-achievement, and increased physical ability. They believed that competing with other teams motivated them to hone their skills and techniques. In particular, when they competed with teams from other ethnic groups, they exerted significant effort to improve their sports-related skills and techniques.

Some of the major personal benefits obtained by participants were improved physical strength and fitness. They saw improvements in endurance, flexibility, muscular strength, and agility through participation in sports-club activities. Most of the female participants said they had negative body images due to lack of exercise—before joining a sports club. However, since they had begun to continually practice and participate in sports, they had seen increased physical function, kept in good shape, and enhanced their body images.

Participants indicated that their challenges with adapting to a new environment had induced low self-esteem and self-confidence. However, by participating in activities with in-group members, they regained their self-esteem and self-confidence. One aspect of this outcome was that they improved their skills and techniques related to activities, and their team members provided practical suggestions and feedback on how to improve them. For example, Yo (a 34-year-old female participant) said:

I aspired to gain advanced table tennis skills and practiced with my team members on most days. I did not realize that I had a talent for table tennis in this way and demonstrated good skills and techniques when I played. As a result, I had more positive emotions and confidence as well.

While most participants put in considerable effort to acquire skills and techniques, they also encountered challenges (such as fatigue, injuries, and competition) while engaging in physical activities. Despite these challenges, they continued to pursue their personal goals regarding sports activities. They used similar phrases to describe their views in this regard: “I never gave up” and “Despite some difficulties, I kept working to achieve my goals.” They felt that they developed the ability to persist throughout difficulties and developed mental toughness.

Kim-J (a 34-year-old male participant) said, “I have lost many games, but I put in the effort required to pursue a variety of different strategies.” He also said he reevaluated strategies he was applying to his game and improved on his positive attributes, which in turn helped him cope with game situations and other areas of his life.

Some participants described that engagement with sports had influenced their lifestyles in a positive way. It appeared that their serious engagement in sports helped them modify their behaviors (e.g., living healthier lifestyles). Won (a 64-year-old male participant) explained how sports-club activities helped him overcome his drinking problem and engage in a healthier lifestyle:

I had a very difficult time adjusting here as an immigrant. I was dependent on alcohol to deal with life … When I became a member of the badminton club, I spent most of my time playing with my team members, including my wife. I even played indoor games at home with my wife. I feel like I turned over a new leaf. When I demonstrated my advanced skills, it made me feel great, and I gained confidence by playing with others.

As these examples indicate, participants regained their self-esteem and started feeling more positive emotions by playing sports. Serious engagement in sports was a positive influence on participants’ lifestyles and fostered healthy behaviors.

## Discussion

This qualitative study was intended to capture health-related benefits of serious engagement in sports—with the background of serious-leisure theory. A few studies have explored serious-leisure experiences among members of ethnic minorities, such as Korean-American males and Korean international students (Heo & Lee, 2007; Lee et al., 2011). The findings of our study broadened the understanding of serious leisure by identifying health benefits among Korean immigrants. The participants (i.e., Korean immigrants) exhibited qualities of serious leisure, such as perseverance, significant effort, the creation of unique cultural worlds, cultural membership and identity, and positive benefits.

Prior studies have provided evidence that immigrants use participation in leisure activities as a coping strategy and resource that facilitates health benefits (Kim, 2012; Kim, Suh, Kim, & Gopalan, 2012). This study expanded the idea that Korean immigrant participants coped well with acculturative stress—in the context of serious leisure. From the psychological aspect of their engagement, Korean immigrants found a psychological comfort zone through participation in sports-club activities, because such activities enabled them to follow their own cultural practices, values, and beliefs. By retaining their cultural identity and membership, Korean immigrant participants could enhance their social and psychological well-being.

The findings of this study are aligned with those of previous studies, which found that among Korean immigrants feelings of belonging and social and emotional support among Korean immigrant participants developed because of cultural membership and cultural identity (Elling et al., 2001; Walseth, 2006). The present study suggests that sports participation, such as serious leisure, produces rich opportunities for participants to build feelings of belonging and social support. In addition, this study indicates that participants create and develop a sense of ethnic bonding and strength through sports participation. It also appears that ethnic bonding—and a shared cultural identity among Korean-immigrant participants—influenced their attitude about sports and competition.

Our study raised further questions about both in- and out-group involvement in sports-club activities. The research supports previous findings that members of ethnic minorities gain health-related benefits when they engage in activities with members of their own ethnic group (Kim et al., 2014; Sanchez & Garcia, 2009). Because of cultural identity and pride, Kim et al. (2014) also found that participation in activities with in-group members served as a motivator for leisure participation. However, other studies have emphasized the value of leisure engagement with other ethnic groups (i.e., out-groups) to facilitate acculturation, as well as improved cultural and ethnic understanding among immigrants (Kim, 2012; Suh & Kim, 2011).

Although this study shows that in-group participation has positive outcomes, it could be argued that this type of involvement needs to be balanced with positive opportunities to participate with out-group members. Several researchers (e.g., Krouwel, Boonstra, Duyvendak, & Veldboer, 2006) have highlighted that intergroup contact between host individuals and immigrants in leisure contexts leads to negative consequences, such as violence and aggression. These studies indicated that any tendency of individuals to participate in leisure activities with their own ethnic groups may result in ethnic separation in a leisure setting. In this study, we intended to stress that in-group leisure participation (as well as out-group leisure involvement) should be facilitated to maximize intragroup bonding, acculturative coping with stress, and personal benefits.

The findings of the current study were consistent with previous studies that serious involvement in sport activities produces positive personal and social outcomes, such as increased confidence and self-esteem, a sense of achievement, acquisition of advanced skills, social relationships, intimate friendships, and expanded social networks (Kim et al., 2014; Mackellar, 2009; Shipway & Jones, 2007). Our study adds to the current body of knowledge: engagement in sports and leisure in a serious manner produces personal and social benefits for Korean immigrants.

This study had some limitations. First, it analyzed and articulated serious-leisure benefits among the participants. Activities pursued by the participants (described as *serious leisure* in this study) were determined based on previous definitions in the literature (Heo & Lee, 2007; Lee et al., 2011). In addition, this study did not explore levels of serious leisure among Korean immigrant participants, although previous studies have suggested a relationship between the level of serious-leisure engagement and health benefits (Kim et al., 2011).

Therefore, it would be beneficial for future researchers to investigate the relationship between the level of serious leisure and health benefits obtained by immigrants. Future researchers also need to address the relationship between serious leisure, acculturation, and health benefits experienced by immigrants. Immigrants may experience different levels of acculturation, which differently affect the value of sports opportunities and consequent health benefits. Finally, to understand immigration and acculturation experiences related to leisure activities other than sports, future researchers might focus on other ethnic groups.

Despite these limitations, this study provides evidence that participants used sports as a way of coping with acculturative stress, creating ethnic strength, and gaining personal benefits. Korean immigrants who pursued engagement in sports-club activities experienced serious leisure. These sports pursuits helped them retain their cultural identity and membership and improve intragroup bonding and cohesion. The resulting health benefits were physical, emotional, and social health.
